# Mesenchymal Stem Cell-Derived Extracellular Vesicles as Idiopathic Pulmonary Fibrosis Microenvironment Targeted Delivery

**DOI:** 10.3390/cells11152322

**Published:** 2022-07-28

**Authors:** Lu Sang, Xiaoqin Guo, Haojun Fan, Jie Shi, Shike Hou, Qi Lv

**Affiliations:** 1 Institute of Disaster and Emergency Medicine, Tianjin University, Tianjin 300000, China; sanglu@tju.edu.cn (L.S.); guoxiaoqinlet@tju.edu.cn (X.G.); fanhj@tju.edu.cn (H.F.); jie.shi@tju.edu.cn (J.S.); 2 Tianjin Key Laboratory of Disaster Medicine Technology, Tianjin 300000, China

**Keywords:** mesenchymal stem cells, extracellular vesicles, idiopathic pulmonary fibrosis, microenvironment, bioengineering, targeted delivery

## Abstract

Idiopathic pulmonary fibrosis (IPF) affects an increasing number of people globally, yet treatment options remain limited. At present, conventional treatments depending on drug therapy do not show an ideal effect in reversing the lung damage or extending the lives of IPF patients. In recent years, more and more attention has focused on extracellular vesicles (EVs) which show extraordinary therapeutic effects in inflammation, fibrosis disease, and tissue damage repair in many kinds of disease therapy. More importantly, EVs can be modified or used as a drug or cytokine delivery tool, targeting injury sites to enhance treatment efficiency. In light of this, the treatment strategy of mesenchymal stem cell-extracellular vesicles (MSC-EVs) targeting the pulmonary microenvironment for IPF provides a new idea for the treatment of IPF. In this review, we summarized the inflammation, immune dysregulation, and extracellular matrix microenvironment (ECM) disorders in the IPF microenvironment in order to reveal the treatment strategy of MSC-EVs targeting the pulmonary microenvironment for IPF.

## 1. Introduction

Idiopathic pulmonary fibrosis (IPF) is a chronic fibrotic interstitial lung disease (ILD) of unknown etiology. It is a rare disease, but it affects millions of people worldwide. It is often associated with fatal outcomes, and the best treatment option remains a lung transplant [1,2]. The characterization of IPF is that an excessive connective tissue depositing by fibroblasts secreting collagen at the injury site, which lasts aberrantly longer and has a wider range than normal wound healing, finally leads to a diffuse deposition of connective tissue in the lung. Extensive scarring in the lung [3] hinders the gas exchange between the alveoli and pulmonary blood vessels, resulting in a disruption of lung function [4], eventually leading to respiratory failure and death. By far, except for lung transplantation, the Food and Drug Administration only approved pirfenidone [5,6,7] and nintedanib [8] as treatment drugs for IPF, while they have many adverse events, such as nausea, rash, and diarrhea [9]. Therefore, it is urgent to explore the pathophysiological mechanisms of IPF and discover new targeted treatment strategies.

In the lung, the proliferating tissue cell at the pathologic center, resident and infiltrating cells, immune cells, cytokines, and the extracellular matrix proteins define the lung microenvironment. In a normal state, they maintain a state of homeostasis. In IPF, both the innate and adaptive immune systems are involved in the development of fibrosis [10]. Histological characteristics in the lungs of patients with IPF are fibroblast foci formation and an excessive deposition of extracellular matrix (ECM) proteins in the fibrotic area [10,11]. An abnormal ECM in fibrotic lungs alters the behavior of epithelial and mesenchymal cells. IPF pathogenesis is associated with the epithelial to mesenchymal transition (EMT). An abnormal EMT also contributes to the myofibroblasts’ formation in the lung interstitium [12,13,14,15].

A variety of mechanisms have been or are currently being studied in IPF. Many predisposing factors, e.g., genetic, epigenetic, and gerontologic, metabolic dysfunction, senescence, aberrant epithelial cell activation, and dysregulated epithelial repair, together with recurrent alveolar epithelial cells (AECs) injury, may occur in IPF. There are multiple genetic variants also associated with an increased risk of IPF development [16,17,18]. Lung development-related genes, such as WNT [19] and sonic hedgehog (SHH), are involved in the driving and process of IPF, as well [20]. Senescent Type II alveolar lung cells (AT2 cells) secrete various cytokines and chemokines, including interleukin 4 (IL)-4 and IL-13, which could stimulate the profibrotic phenotypic changes in alveolar macrophages and lead to persistent inflammation [21]. Moreover, in a p53-dependent cellular senescence model, senescence rather than a loss of AT2 cells promotes spontaneous, progressive pulmonary fibrosis [22].

Recently, studies have suggested that microbiota in the airways of patients with IPF correlate with increased alveolar profibrotic cytokines [23]. Lung dysbiosis precedes peak lung injury and can promote alveolar inflammation and aberrant repair [23]. In fibroblastic foci, AECs overexpress c-MET, a receptor tyrosine kinase that can activate a wide range of signaling pathways involved in cell proliferation, motility, migration, and invasion, which potentially interferes with the EMT and fibroblastic foci formation in IPF [24,25]. Mitochondrial dysfunction and the generation of mitochondrial reactive oxygen species lead to senescence and the activation of phosphoinositide 3-kinase (PI3K)/protein kinase B (Akt) signaling [26]. The deregulation of the PI3K/AKT signaling pathway has been associated with suppressing inflammatory cell infiltration, producing inflammatory cytokines and preventing pulmonary fibrosis [27,28]. TGF-β1 plays a key role in tissue fibrosis by promoting collagen deposition, the EMT process, and regulating the proliferation and apoptosis of different cells [29].

Mesenchymal stem cells (MSCs) are self-renewing pluripotent stem cells derived from a wide range of sources. Current studies suggest that MSCs transplantation can be an effective way to repair IPF [30,31]. However, the application potential of MSCs is limited by their low survival rates after transplantation into the target organs, susceptibility to embolism and risk of tumor formation [32,33]. Reports have suggested that MSCs may have a fibroblastic or pericytic origin. Experimental data suggested that MSCs may have a profibrotic microenvironment potential effect on IPF [34]. In recent years, the adopted viewpoint demonstrated that EVs exert an important role in the paracrine/endocrine actions induced by MSCs therapy [35]. The administration of mesenchymal stem cell-extracellular vesicles (MSC-EVs) can promote protective actions similar to those of the MSC parent cells [36,37]. Studies showed that EVs carry many cargos, such as DNA, proteins, lipids, mRNA, miRNAs, siRNA, and non-coding RNA [38]. EVs not only act as messengers among cells but also as effectors affecting the microenvironment. In general, intravenously administrated EVs are cleared by macrophages in the mononuclear phagocytic system (MPS) and preferentially aggregate in MPS organs, such as the lung, liver, and spleen [39]. These indicate that we should enrich EVs and target the damaged lung tissue for a sufficient duration of action to increase treatment efficiency.

Nowadays, exploring strategies for delivering EVs-targeted therapy to the lung is critical. Recent studies have demonstrated that the targeted delivery of transplanted MSC-EVs to the focal lung site can be achieved by multiple means [40], including intravenous injection and nebulization, in combination with biotechnology, such as the functional modification or as a delivery vector, etc. This review elaborates on the role of the inflammatory milieu, the immune system, and ECM in IPF, and discusses the therapeutic effect of MSC-EVs on IPF based on their effect in the pulmonary microenvironment. These insights will contribute to the development of the therapeutic value of MSC-EVs for IPF.

## 2. Characteristics of EVs

EVs are small membranous vesicles composed of a phospholipid bilayer secreted by various cells [41], which contain cargos of proteins, nucleic acids, and lipids. Thus far, cells essentially release three EV subtypes, separated by centrifugation: exosomes, microvesicles (MVs) and apoptotic bodies, with diameters of 20–150 nm, 50–1000 nm and greater than 1000 nm, respectively [42]. The characteristics of EVs are summarized in Table 1. According to a worldwide International Society for Extracellular Vesicles (ISEV) survey, differential ultracentrifugation was the most commonly used primary EV separation and concentration technique, with various other techniques, such as density gradients, precipitation, filtration, size exclusion chromatography, and immunoisolation [43].

**Figure 1:**
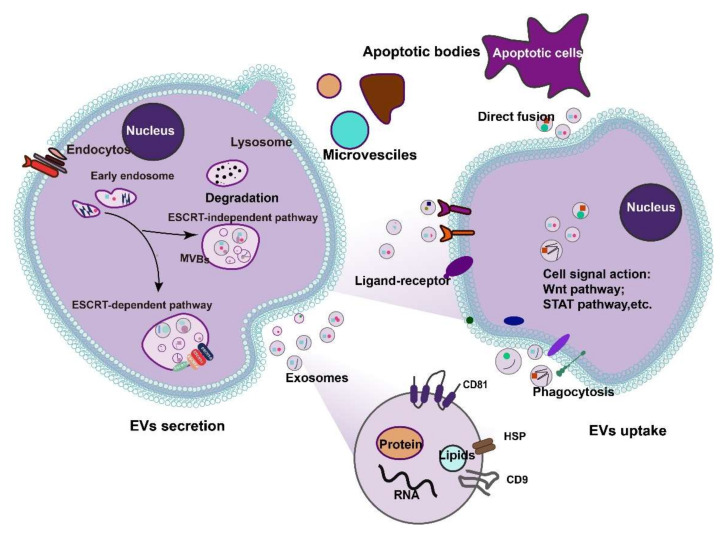
Secretion and uptake of extracellular vesicles (EVs). EVs are currently classified into three categories depending on their origin, secretion mechanism, and size: exosomes, microvesicles, and apoptotic bodies. EVs interact with recipient/target cells and act as messengers for signal delivery: interactions with plasma membrane receptors, phagocytosis into cells, and direct fusion with the plasma membrane.

EVs-mediated intercellular communication depends on the origin of the extracellular vesicles and the source of recipient cells. Once released, EVs can correspond to the recipient cells via ligand-receptor interaction or through the vesicle internalization by phagocytosis, endocytosis, or direct membrane fusion [44]. EVs modulate cellular responses by transferring the biomolecular cargo it carries, such as proteins, lipids, mRNA, and microRNA, resulting in signaling cascades that alert or trigger biological processes in target cells [45]. This allows vesicles to interact with selected target cells locally or at a distance and perform specific biological functions. The specific sorting machinery of MVBs could specify the final fate of MVBs between exosome secretion and lysosomal degradation [41,46] (Figure 1).

## 3. Microenvironment in IPF

**Figure 2:**
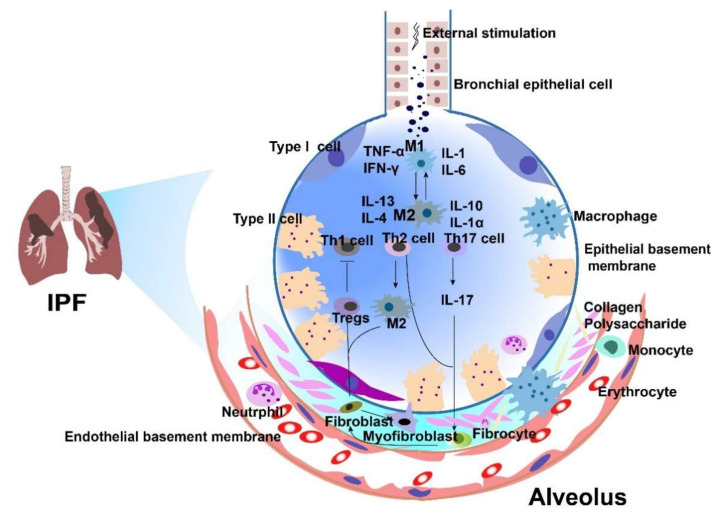
The dyshomeostasis of the microenvironment in idiopathic pulmonary fibrosis (IPF). In individuals with IPF, lung epithelial injury leads to the production of inflammatory factors, profibrotic cytokines, and chemokines secreted by alveolar macrophages. This results in activation of fibroblasts and differentiation into myofibroblasts, which produces extracellular matrix (ECM), leading to thickening of the lung interstitium. In IPF, the microenvironment altered the Th1/Th2 balance in the lung. IFN-γ produced by Th1 cells inhibits fibrocyte differentiation and promotes M1 macrophage formation. IL-17 production by Th17 cells in the lung also promotes fibroblast activation. The imbalance between Th17 and Tregs can affect pulmonary fibrosis.

When the healing process of damaged tissues in IPF is out of control and excessive, it will lead to tissue remodeling, abnormal scar tissue formation, and fibrosis [47]. The main site of pathological pulmonary fibrosis is the interstitium and includes the alveolar epithelium, pulmonary capillary endothelium, basement membrane, and perivascular and lymphatic tissues. In this review, we summarize the pathogenesis of IPF (Figure 2).

### 3.1. Inflammatory Microenvironment in IPF

IPF can be considered a chronic inflammatory process. The role of inflammation, as an important component of the etiology of IPF, is controversial and is sometimes regarded as an incidental phenomenon of fibrosis. The inflammatory hypothesis originates from mild immune infiltration and increased lung pro-inflammatory cytokines in IPF [48,49,50]. Repeated exposure of lung epithelial cells to a toxic environment can disrupt the epithelial barrier and ultimately lead to airway pathology [51]. Damage to AECs activates macrophages, dendritic cells, and other innate immune cells, further leading to inflammatory immune responses. When Type I alveolar lung cells (AT1 cells) are damaged or missing, the AT2 cells proliferate and transdifferentiate into AT1 cells [52]. In pathological conditions, AEC and immune cells (mainly alveolar macrophages) could release inflammatory factors, such as IL-6, tumor necrosis factor (TNF-α), interferon-gamma (IFN-γ), chemokines, etc., and recruit fibroblasts to accumulate to the injured area and differentiate into myofibroblasts that secrete collagen and proteins. Many profibrotic factors, such as the connective tissue growth factor (CTGF), transforming growth factor 1 (TGF-β1), and fibroblast growth factor (FGF), can cause the excessive secretion of collagen and then deposition in the lung interstitium, constantly forming “scars” in the lung, resulting in pulmonary fibrosis [53,54]. The stimulation of lung epithelial cells might disrupt the balance between lung epithelial and mesenchymal cells [52,55]. External harmful substances stimulate pulmonary endothelial cells to secrete inflammatory factors and chemokines to induce phenotypic transformation of lung endothelial cells [56]. Genes related to inflammation have also been proved to be associated with IPF. A study showed that the gene variants in toll-interacting protein (TOLLIP) could increase a pro-inflammatory response in IPF patients [57]. The suppression of chronic inflammation can turn off pulmonary fibrogenesis in nonspecific interstitial pneumonia, which demonstrates that inflammation is a possible pathological mechanism in lung fibrosis [58,59].

In past clinical studies, many anti-inflammatory trials have been conducted in an attempt to treat IPF [60]. Trials with anti-TNF-α drugs and prednisone did not achieve the expected results [61,62]. Additionally, antibodies to the anti-inflammatory cytokine IL-13 have entered phase 2 clinical trials in IPF but did not show definite results [63,64]. Antibodies against IL-4/IL-13 have failed to demonstrate efficacy in IPF patients [50,51]. However, anti-IL-13 trials showed some positive effects on IPF rates [65]. Therefore, we hold the view that the hypothesis that the inflammation mechanism alone participates in the impact of IPF is not comprehensive and not feasible as a single treatment strategy.

### 3.2. Immune Dysregulation in IPF

Histopathological samples of IPF, obtained from susceptible older adults, showed little evidence of inflammation [60,63]. Some other studies, which reported treatment with only anti-inflammatory agents, seemed to have no effect on IPF [47,50]. These clinical observations strongly suggested that inflammation was an important but optional event and that other mechanisms may exist, as immune modulation coexists in pulmonary fibrosis. In contrast, a study of a mouse model of pulmonary fibrosis has also reported that immunomodulatory processes other than inflammation promoted the development of fibrosis [66]. The immunomodulatory system is critical for maintaining immune homeostasis in IPF.

#### 3.2.1. Innate Immune Response

Alveolar macrophages (AMs) are directly stimulated by various external complex factors and participate in the construction of the first line of defense against external invasion. Macrophages, one of the most abundant immune cell types in healthy lungs, are the major innate immune cells in lung homeostasis and play a central role in tissue repair and immunity. Alveolar macrophages play a key role in pulmonary homeostasis by clearing apoptotic cells and debris, regulating wound healing, scavenging microbes, eliminating inflammation, and helping to initiate immune responses to pulmonary pathogens.

Macrophages are involved in the regulation of immune homeostasis through two types of polarization, namely M1-type polarization (classical activation) and M2-type polarization (alternative activation). Environment changes and molecular mediators can transform macrophages from M1 to M2 [67,68,69]. IFN-γ, lipopolysaccharide (LPS) and other pro-inflammatory factors in the microenvironment induce the M1 polarization of macrophages, and they produce and secrete large amounts of pro-inflammatory cytokines, such as IL-12, TNF-α and IL-6, which play a role in killing pathogens, foreign bodies and removing endogenous abnormal tissues and cells in the immune microenvironment, thus maintaining tissue inflammation [70]. If the M1 macrophages’ response is not effectively controlled, tissue damage will continue. The M2 macrophages are usually induced by IL-4 and IL-10 in the microenvironment. By releasing inflammatory cytokines, such as IL-10 and TGF-β1, they prevent excessive inflammatory reaction damage, promote the repair of tissue damage, further promote the proliferation of fibroblasts and make them secrete collagen, and thus, promote the progression of pulmonary fibrosis. The imbalance of the polarization of macrophages can disrupt the microenvironmental immune homeostasis and become the initiating factor of an inflammatory storm and over-repair *in vivo*.

Alveolar macrophage is one of the important factors that determine the course and outcome of IPF. M2 macrophages have multiple functions and play a critical role in asthma, interstitial lung disease, and cancer. M2 macrophages are widely present in the fibrotic lung [68]. They can secrete many growth factors, including TGF-β, FGF, platelet-derived growth factor-α (PDGFα), insulin-like growth factor 1 (IGF1) and vascular endothelial growth factor (VEGF) [71]; anti-inflammatory and immunosuppressive mediators, such as IL-10 and TGF-β1, specific chemokines (CCL17 and CCL22, C-C motif) [72]. Therefore, macrophages are closely related to the regulation of immune responses. In IPF patients, the increased concentration of CCL18 in bronchoalveolar perfusion fluid (BALF) (produced by M2 macrophages) suggested that the macrophage driving mechanism is involved in IPF [72]. In addition, CCL18 can attract T cells to the lung and activate fibroblasts [73]. M2 macrophages also contribute to ECM formation by up-regulating the L-arginine metabolism [70]. However, another study also indicated that M2 macrophages in humans had been reported to degrade ECM components by secreting matrix metalloproteinases (MMPs), leading to a reduction in fibrosis [74]. In conclusion, the function of macrophages is highly dependent on the local environment, and macrophages have vagaries functions of anti-fibrosis, promoting fibrosis and tissue regeneration.

#### 3.2.2. Adaptive Immune Response

T cells are widely present in active disease areas of IPF patients [75,76,77]. In IPF patients, the CD4+ and CD8+ T cells in the lungs and BALF increased [78]. In T-cell-deficient mice, bleomycin exposure can cause less ECM formation and fibroblast proliferation inhibition, leading to pulmonary fibrosis [79]. The function of T cells depends on their different phenotypes—T cell subsets have profibrotic, antifibrotic potential or no role at all. Recent evidence suggests that helper T (Th) cells (Th1 cells, Th2 cells, Th17 cells, Th9 cells, and regulatory T cells (Tregs)) and B cells play important roles in the pathogenesis of IPF.

The ratio of Th1 and Th2 T cells in the lung may determine the course of lung injury and the progression of its fibrosis [80]. An unbalanced Th1/Th2 immune response is considered to be the key pathogenesis of IPF. IFN-γ, produced by Th1 cells, inhibits fibrocyte differentiation and promotes M1 macrophage formation [67,81]. Hence, the Th1 cells may protect the lung from fibrosis [82,83]. However, clinical trials reported that the use of recombinant IFN-γ or anti-IL-13 monoclonal antibodies failed to show any clinical benefit in patients with IPF [63,84].

The profibrotic Th17 cells can produce IL-17(A), IL-21 and IL-22 to promote tissue inflammation. IL-21 promotes the primary alveolar macrophages toward the M2 phenotype polarization in pulmonary arterial hypertension (PAH) [85]. The administration of recombinant IL-21 reduced the Th1 and Th17 responses and aggravated chlamydial lung infection in mice [86]. One literature showed the active area of IPF was infiltrated by IL-17 [76]. Further, MSCs overexpressing TGF-β1 can regulate lung inflammation and alleviate lung injury by regulating the Th17/Treg imbalance in the lungs of acute respiratory distress syndrome (ARDS) mice [87]. An imbalance between Th17 and Tregs can affect pulmonary fibrosis [88,89]. Thus, we believe that the balance between the Th17 cells and Tregs is critical in the development of lung fibrosis.

Tregs may be detrimental in the early stage of pulmonary fibrosis in mice; however, they have a protective effect in the late stage of pulmonary fibrosis [90]. Studies have shown that Tregs levels are reduced in the blood and BALF of IPF patients [91]. Tregs depletion can increase IFN-γ and decrease Th17 cells level after bleomycin administration and ameliorate lung fibrosis [92]. Additionally, Tregs can resolve fibroproliferation by reducing fibrocytes recruitment depending on blockade of the chemokine (C-X-C motif) ligand 12 (CXCL12)-CXCR4 axis [93]. But it’s also been documented that Tregs can promote EMT through β-catenin accumulation in lung epithelial cells, and accelerate radiation-induced pulmonary fibrosis [94]. Their functions and variation tendency depend on the course of lung fibrosis [95]. These reports revealed that Tregs might have a two-way action in antifibrotic and profibrotic.

Similar to the T cells, the number of B cells is increased in the lungs of IPF patients. Generally, B cells within tertiary lymphoid structures (TLOs) are responsible for local antibody production [96]. Elevated CXCL13 concentrations were found in the lungs and serum of IPF patients, and this can chemoattract B cells to B cell follicles in the IPF lung [97]. B regs (regulatory B-cells) can inhibit the proliferation of Th1 and Th17, downregulate the differentiation of dendritic cells (DCs), and promote the expression and differentiation of FOXP3+ Tregs. IL-10 production by B cells can produce anti-inflammatory immunoglobulin G (IgG) 4 and increase Tregs activity, and modulate the progress of lung inflammation and fibrosis [98,99]. Autogenic immunoglobulin is found in the majority of IPF patients [100]. Mesenchymal stromal cells, derived from the human amniotic membrane (hAMSCs), have contributed to reducing the progression of fibrotic lesions by modulating the B-cell response, which may increase lung expression of the Tregs marker, Foxp3, and induce macrophage polarization to an anti-inflammatory phenotype (M2) [101].

In conclusion, the studies on the role of T cells in IPF suggest that single T cells are unlikely to be sufficient to treat IPF immune disorders, and future research should focus on how to maintain the internal homeostasis of T-cell subtypes after lung tissue injury. A more detailed and comprehensive understanding of the immune system’s role in IPF may lead to the development of treatments for IPF.

### 3.3. ECM Microenvironment in IPF

The lung extracellular matrix (ECM) consists of the basement membrane (BM), collagen, glycoproteins, and proteoglycans. ECM has complex network structures. ECM is the main scaffold that supports the tissue structure, the lung’s general mechanical stability and elastic retraction. In addition, ECM stores various growth factors and cytokines. Fibrosis is a normal, indispensable process to protect wound healing for the host body. During the normal wound healing process, myofibroblasts can produce ECM proteins and large amounts of collagens, and once the active phase of repair is finished, the myofibroblasts quickly disappear via apoptosis [102]. In IPF, however, these cells are anti-apoptotic, perpetuating and nourishing the fibrotic process, leading to excessive deposition of ECM. Studies have shown that ECM can affect biological processes, such as cell differentiation, proliferation, adhesion, morphogenesis, and phenotypic expression. IPF is characterized by progressive fibroblast proliferation and extensive ECM deposition, resulting in alveolar structure destruction and pulmonary parenchyma remodeling [103]. The ECM in IPF may enhance pathological cross-linking, which can promote fibroblast growth and increase resistance to normal ECM turnover [104].

Activated fibroblasts and myofibroblasts are key effector cells of fibrosis [102]. Fibroblasts are one of the main ECM-producing cells and can promote the harmful remodeling of IPF lung tissues [105]. A number of fibrocytes were found in the fibrotic-active region of IPF [106]. Fibrocytes are the progenitors of collagen-secreting fibroblasts and may contribute to the development and accumulation of fibroblasts [107]. Fibroblasts continuously release recruitment factors in the fibrotic environment, which enable inflammatory cells to continuously go to the site of injury. While myofibroblasts, newly formed after injury, could not be found in normal lung tissue and can produce a large amount of collagen and other ECM proteins to promote ECM [102].

Although the exact mechanism of the occurrence and development of abnormal fibrosis remains unclear, the tissue microenvironment has become a key point in understanding the chronic and progressive nature of fibrosis.

## 4. MSC-EVs Mediated Homeostasis on IPF

The paracrine factors of MSCs are packaged within MSC-EVs. MSC-EVs can maintain a similar therapeutic effect to MSC and low immunogenicity. The MSC exosome is not a single drug or antibody but a comprehensive biological therapy, which can play a targeted therapeutic role, increase accuracy, and target pathological sites. Preclinical studies have shown that MSC-EVs showed anti-fibrosis and promoted tissue repairing effects in a variety of fibrosis diseases, such as lung [108], liver [109], kidney [110], heart [111,112], and skin [113]. The MSC-EVs clinical trials have been progressively explored in recent years, but the application is still in the initial stages. According to the National Institutes of Health registered clinical trials, the MSC-EVs application mainly included ARDS (NCT05127122), coronavirus disease 2019 (COVID-19) (NCT04657458, NCT05125562, NCT05116761, NCT04493242, NCT05354141), Type 1 diabetes (NCT02138331), cardiovascular disease (NCT02017132), dystrophic epidermolysis bullosa (NCT04173650), and acute ischemic stroke (NCT03384433). Clinical trials in IPF lack quantity in the concerned study. Of note, one clinical trial involving MSC exosomes in the treatment of COVID-19 together with pulmonary fibrosis is currently being recruited (NCT05191381) [114]. In summary, the MSC-EVs therapy for IPF is full of hope, and more attention should be paid to it in the future.

### 4.1. MSC-EVs Plays an Anti-Inflammatory Role in IPF

MSCs are also known to communicate with other cells by secreting EVs [115]. Mansouri et al. found that human bone marrow mesenchymal stem cell (BMSC) exosomes effectively alleviated the bleomycin-induced core features of pulmonary fibrosis and lung inflammation [108]. In their study, it was also shown that MSC exosomes could modulate lung macrophage phenotypes, shifting the lung pro-inflammatory/classical and nonclassical monocytes as well as the alveolar macrophages toward the monocyte/macrophage profiles. They believed that BMSC exosomes respond to their microenvironment by releasing proteins or miRNAs, and the contents of the inflammatory environment will contain the properties of inhibiting pulmonary fibrosis. Another study showed that mice receiving human umbilical cord Wharton’s jelly (WJMSC) or BMSC exosomes could ameliorate collagen deposition, reduce inflammation and restore lung function through macrophage immunomodulation in experimental bronchopulmonary dysplasia [116]. The exosomes derived from human amnion epithelial cells (hAECs) modulated neutrophil myeloperoxidase in a bleomycin challenge and reduced lung inflammation [117].

### 4.2. MSC-EVs Can Modulate the Immune Response in IPF

Although widespread suppression of inflammation in IPF may be harmful, that does not mean the immune system is not involved in the cause or treatment of the disease. A large number of studies have shown that the immune system plays a role in IPF by modulating the activation and function of fibroblasts. Genetic studies have shown that immune responses play an important role in IPF, which is associated with polymorphisms in immune-related genes that encode toll-like receptor 3 (TLR3), TOLLIP, and IL-1 receptor antagonists (IL-1RA), and with increased disease risk or severity [57,118,119,120]. Therefore, we believe that the development of targeted immunomodulatory therapies can change the course of IPF disease.

A growing amount of literature suggests that MSC exosomes can improve immunomodulatory functions in IPF. Studies have shown that human BMSC-EVs can modulate the activity of the cells involved in the immune response. Shentu et al. demonstrated that bone marrow-derived MSC exosomes could inhibit TGF-β1-induced myofibroblast differentiation, and the mechanism depended on blocking the interaction of Thy-1 with beta integrins, which prevented the suppression of myofibroblast differentiation [121]. Another study showed that hAEC exosomes could increase AT2 cells proportions. Moreover, immunomodulatory effects were observed to reduce the percentage of CD4+T cells and interstitial macrophages in the lung, and finally, they concluded that hAEC exosomes are able to directly regulate the function of neutrophils, macrophages, and T cells [117].

### 4.3. MSC-EVs Regulate ECM Homeostasis in IPF

It is well known that the cellular environment and ECM determine the behavior of cells, such as migration, proliferation, and differentiation. The activation of EMT promotes the generation of fibrotic cells, and polarized epithelial cells undergo molecular and phenotypic changes that enable them to attain mesenchymal and undifferentiated potential. Such plasticity changes mean that cells acquire an enhanced ability to produce ECM components on the one hand and become more motile and more invasive on the other [122,123]. Bleomycin-induced pulmonary fibrosis can be reversed by reducing the collagen deposition after intravenous BMSC-exosome treatment [108]. Another study showed that hAEC exosomes could reduce α-SMA and pulmonary collagen and inhibit the differentiation of myofibroblasts [117]. Analyses revealed that MSC-exosome treatments could attenuate and resolve bleomycin- and silica-induced fibrosis by reestablishing a normal alveolar structure and decreasing both collagen accumulation and myofibroblast proliferation [124]. In addition, BMSCs EVs inhibited the TGF-β-induced differentiation of normal and IPF fibroblasts into myofibroblasts by decreasing the expression of α-SMA, fibronectin, and type III collagen [125]. Sun et al. evaluated the protective potential of the exosomes derived from menstrual blood stem cells (MenSCs) by reducing the collagen deposition and regulating the ROS activity on alveolar epithelial cells through transporting miR-let-7 in an IPF model [126].

## 5. MSC-EVs Treatment Administration for IPF

**Figure 3:**
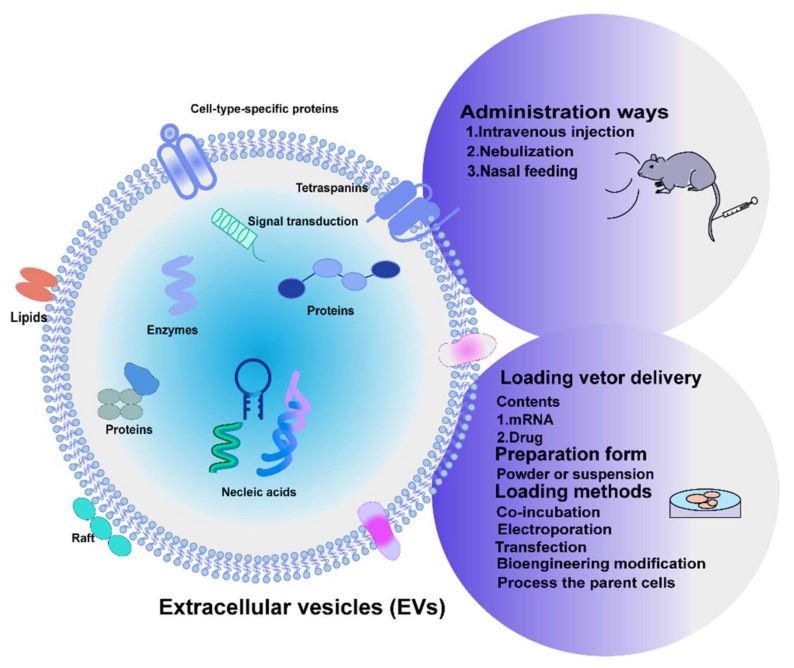
Overview of therapeutic modalities of MSC-EVs for IPF. Administration routes in EVs-based strategies applicable in IPF. MSC-EVs loading techniques to produce target and effective EVs-based nanotherapeutics.

MSC-EVs are now considered a more effective and safer alternative to MSC transplantation and have shown therapeutic benefits in many preclinical models of lung injury and disease. In fact, EVs have characteristics that make them ideal drug delivery systems because of their high stability and low immunogenicity in the blood. In addition, the inherent homing ability of EVs to the injury sites can make them avoid off-target side effects and enhance a specific uptake of target cells. Therefore, there is great potential for the development of MSC-EVs therapies. In fact, the clinical transition to MSC-EVs is still in its infancy and needs to face many challenges. Here, we summarize the studies of MSC-EVs administration in pulmonary fibrosis *in vivo* in Table 2 [20,108,117,124,125,126,127,128,129,130,131,132,133,134,135,136,137,138,139] and an overview of therapeutic modalities of MSC-EVs for IPF in Figure 3.

### 5.1. Systemic Delivery of MSC-EVs

The pulmonary distribution and subsequent uptake of EVs are the bottleneck problem for its therapeutic effect. In preclinical studies, intravenous administration is the most commonly used mode of administration. Intravenous injection has a faster onset of action and higher bioavailability. The EVs showed a high blood flow stability and pulmonary distribution after injection [140]. The accumulation of EVs in the lungs is mainly due to the high vascularization of these organs, and eventually, EVs have the ability to home in the event of tissue damage. Preclinical studies have shown that the intravenous injection of human BMSC-EVs has an immunomodulatory effect in reducing monocyte-driven inflammation, preventing and reversing bronchial bleomycin-induced pulmonary fibrosis in mice, thereby improving lung morphology and lung structure and reducing collagen deposition [108,121]. An intravenous pretreatment of high molecular weight hyaluronic acid (HMW HA) enhances the delivery of MSC-EVs to the injured alveolus and reduces inflammation in mice pseudomonas aeruginosa (PA) pneumonia [141].

Moreso, miRNAs have strong tissue specificity, so some of them are closely related to IPF. The anti-fibrosis effect of miRNAs in MSC-EVs has become a research hotspot of IPF. The MSC exosomes contain several miRNAs that can up-regulate the pro-fibrosis genes in IPF fibroblasts. One study showed that the antagonism of miR-let-7 pretreated MenSCs-infused exosomes into the tail vein of mice could reverse the exosome protection against lung fibrosis and mitochondrial DNA damage in bleomycin-exposed lung fibrosis, but it was not completely reversed [126]. Zhou et al. found that human BMSC-EVs pretreated with miR-186 mimics through a tail vein injection could impair the activation of fibroblasts by inhibiting the expression of SRY-related HMG box transcription factor 4 (SOX4) and Dickkopf-1 (DKK1), and ultimately alleviate pulmonary fibrosis [133]. Notably, the quantification of the total exosomes in serum is largely variable; the proteins in MSC exosomes have biological functions like those of RNA in MSC exosomes [142]. MSC exosomal RNA is a highly heterogenous RNA population of approximately 100 nts [143]. Baglio et al. conducted an MSC exosome RNA sequencing analysis, and they identified miRNAs as 2–5% of total RNA [144]. Therefore, quantifying certain microRNAs in clinical samples using standard methods remains a challenge.

### 5.2. Direct Delivery of MSC-EVs to Lung

The solubility and small size of EVs compared to cells make it possible for aerosol inhalation and intranasal infusion. Inhalation is a non-invasive, fast-acting route that allows lower doses to achieve the same effect, avoiding gastrointestinal side effects and even hepatic metabolism, thereby increasing EVs’ efficiency. Currently, intakes of EVs can be included in dry powder or a liquid suspension. An integrated process of ultrafiltration and lyophilization, in accordance with good manufacturing practice (GMP), has been proposed to convert MSC-EVs into a freeze-dried powder, which can be dissolved to produce an EVs suspension [145]. The lyophilization of key components in exosomes does not have much impact, but there are also literature tasks affecting the stability of secreted histones [146]. The evidence showed that, in direct contrast to the intravenous (IV) route, the immunity and matrix regulatory cells derived from human embryonic stem cells (IMRC)-EVs are mainly retained in the lungs for intratracheal delivery and primarily in the liver and spleen when administrated through IV route [139]. Dinh et al. demonstrated the therapeutic effect of the direct atomization of MSC exosomes on bleomycin-induced pulmonary fibrosis rats and confirmed the reduction of pulmonary fibrosis score and pulmonary fibrosis markers, such as α-SMA. However, they did not control the quality and did not mention the quantitation of the exosomes used [124]. They also found that the genes hsa-let-7a-5p and haa-let-7f-5p, which belong to the highly conserved let-7 family, were the most up-regulated in the MSC exosome samples. Let-7 miRNAs have been found to play important roles in biological development, stem cell differentiation and tumorigenesis. It has also been reported that the intranasal infusion of exosomes one day after bleomycin stimulation reduced lung inflammation, while treatment on day seven improved alveolar collapse and reduced fibrosis [117]. Ren et al. reported that the intranasal delivery of human placenta MSC exosomes could substantially enhance lung IL-10 and promote producing more lung interstitial macrophages (IMs), which may originate from the spleen, thus contributing to protecting mice from allergic asthma [147]. Moreover, in the ALI model, a study showed that inhaled WJMSC-EVs outperformed those injected via the tail vein, which not only decreases the pathological scores and promotes lung repair but also shows that the EVs reach lung tissue faster than the tail vein injection [148]. Bandeira et al. also showed that the local delivery of adipose-tissue-derived MSCs (AD-MSCs) exosomes by intratracheal injection ameliorated fibrosis and inflammation, but dose-enhanced EVs yielded better therapeutic outcomes in this silicosis model [129]. However, some researchers have reported that although EVs isolated from MSCs can reduce pulmonary fibrosis, MSCs are more therapeutically effective than EVs [128]. The source and number of EVs isolated from MSCs lack dose-effectiveness measurements.

In conclusion, so far, it is difficult to predict the safety and the appropriate and effective dose of human MSC-EVs, due to the lack of preclinical animal studies outcomes and the lack of a unified consensus on dose standards. Therefore, we believe that the dose, timing, and frequency of MSC-EVs administration, as well as the drug form, need to be further explored.

### 5.3. MSC-EVs as a Targeted Delivery Vector for IPF Treatment

At present, the most advanced research method as a carrier is combined with the method of biological engineering. Exosomes have many of the properties of drug delivery vehicles that make them more attractive than conventional drug carriers, such as their natural nanosized structure, homing effects and low immunogenicity. Engineered exosomes are currently the mainstream of the industry because they can modify their targeting and improve drug loading efficiency. Exosomes derived from MSCs are about 40–80 nm in size and can deliver 20–30 bp microRNAs. Natural vesicles can be considered the most advanced delivery technology, which is not only easy to design and target but can also naturally carry macromolecules. It is expected to make up for the shortcomings of the existing delivery technology and become a unique drug delivery system.

Exosomes can be loaded with bioactive molecules, such as RNA (miRNA [126], siRNA [149]), protein [150], chemical drugs [151], etc. MSC-exosome drug loading technology has been widely used in heart diseases, neuro-diseases and tumors [152,153]. This suggests that EVs, as drug carriers, can play an increasingly important role in targeting the treatment of IPF. Currently, there are few studies on MSC exosomes as a vehicle for the treatment of IPF. Li and his team designed a new hybrid drug delivery system of clodronate (CLD)-loaded liposomes and fibroblast-derived exosome (EL-CLD) containing the macrophage inhibitor CLD, with nonspecific phagocytosis inhibition and fibroblast homing properties [154]. CLD liposomes can effectively reduce the phagocytosis of nanoparticles by the liver after intravenous injection. In addition, the EL-CLD hybrid system preferentially accumulates in the fibrotic lung and further penetrates into the ECM. The drug delivery system was further coated with the anti-fibrosis drug nintedanib, which showed significantly better effects than traditional drug therapy in improving lung function and inhibiting pulmonary fibrosis in pulmonary fibrosis model mice.

Recent studies have reported that functional modifications of exosomes can further enhance the advantages of exosomes. Functionalized modifications of exosomes can prolong the circulation time of exosomes, enhance the delivery efficiency of exosomes in the cytoplasm, and promote stronger targeting of exosomes [155]. In addition, the surface of exosomes can also be modified in non-covalent ways, such as electrostatic interactions, receptor–ligand binding, and hydrophobic reactions [156]. To improve the delivery efficiency of EVs to ischemia-injured myocardium, some researchers modified the MSC exosomes with monocyte mimics through the means of membrane fusion [157]. The monocyte-mimicking bio-inspired BMSC-EVs exhibited an enhanced targeting efficiency to injured myocardium by mimicking the recruitment feature of monocytes following a myocardial ischemia-reperfusion injury (MI/RI). In a nonhuman primate (NHP) myocardial infarction (MI) model, the delivery of miR-486-5p-overexpressing normoxia-preconditioned exosomes can promote cardiac function [158]. Another study designed targeting peptide-Lamp2b fusion proteins to include a glycosylation motif at various positions, and it enhanced the targeted delivery of the exosomes to neuroblastoma cells [159]. Additionally, nanobodies can be anchored on the surface of EVs via glycosylphosphatidylinositol (GPI), thereby altering their cellular targeting [160]. Some researchers encapsulated exosomes with si Clathrin by electroporation to significantly knock down the clathrin heavy chain (Cltc) and decrease the macrophage uptake of the exosomes *in vitro* and *in vivo*, which then prevented MPS-mediated endocytosis in the spleen and liver, thereby increasing intravenous EVs delivery to the heart and other organs [161]. Belhadj et al. also used the combined “eat me/don’t eat me” strategy of selective endocytosing EVs by macrophages, thus reducing the phagocytosis of EVs by macrophages, which increased the tumor area accumulation of EVs by 123.53% compared with the traditional nanocarriers which were without loading CD47-enriched exosomes or drugs [162]. Another study found that a group of tyrosine phosphatase 2 (Shp2) regulated the synthesis and secretion of EVs, elucidating that the phosphatase Shp2 negatively regulates the production of alveolar epithelial exosomes through dephosphorylation modification, which suggested a new pathway of phosphatases involved in the microenvironment of lung inflammation [163]. Park and his colleagues studied the MSC nanovesicles (NVs) produced by disintegrating cells through the continuous extrusion of MSCs, and they indicated that NVs could increase the expression of the IL-10 in sepsis, suggesting that artificial NVs may be novel clinical EV-mimetics for patients [164].

## 6. Challenges for Application

Due to the unknown cause of the disease, the processes driving IPF are complex, and there are no models that can fully explain the pathogenesis of IPF. The bleomycin models are the most commonly used model for IPF research. The observations in both the bleomycin and silica models demonstrated that MSC-EVs inhalation therapy was effective in treating pulmonary fibrosis [124]. Additionally, the anatomical features of the organism caused many obstacles. For example, a bronchial tree with multiple branches and curvature may make EVs unable to reach the lung or become unevenly distributed in lung tissue, deposited in the atmospheric duct or accumulated in capillaries [165]. Macrophages in the alveoli engulf particles or EVs, thereby clearing them before they reach the tissues.

The dose and route of the administration of MSC-EVs vary widely across preclinical animal studies; thus, the safe and effective dose used to treat these pathological conditions remains to be determined. The dosage control problem is mainly its intake dosage rather than inhalation dosage, actually. The delivery of the MSC-EVs via inhalation of human lungs has been studied, including viral pneumonia COVID-19. MSC-EV treatment trials in COVID-19, registered on Clinical Trials (e.g., NCT04276987, NCT04491240, NCT04602442), showed the doses the clinicians used and the availability of the inhalation pathway. However, EVs treatment is not permanent, they may be metabolized, and there is even an argument to the contrary that EVs are not as effective as MSCs. In addition, surfactants in the lower lungs have the possibility of destabilizing liposomes and lipid nanoparticles. As reported by some authors [166,167,168], MSC-EVs may require repeated dosing to maintain their therapeutic potential. Moreover, the accumulation and retention of MSC-EVs in different organs need to be considered. The distribution of EVs to the heart is limited by the rapid clearance of MPS from the blood and subsequent accumulation in the liver and spleen [169]. It can be seen that the excessive retention of EVs in the liver during intravenous administration not only affects their bioavailability but also brings the risk of liver damage. The biodistribution of EVs influences the therapeutic efficacy and toxicity after systemic administration [170]. Similar to any other nanotherapeutics, unmodified exosomes were delivered systemically in animals, accumulated preferentially in the liver, kidney, and spleen, and were rapidly eliminated by biliary excretion, renal filtration, or phagocytosis in the reticuloendothelial system, respectively.

Then, when MSC-EVs can actually be used in clinical treatment, sterility is also a question. According to the small volume of EVs, filtration sterilization can be carried out at the end of the separation process [171]. Quality control is required to ensure sterility, pyrogen, and the absence of foreign viruses. Biological safety, such as hepatorenal toxicity, survival rates, and adverse reactions, requires a comprehensive and long-term evaluation [124]. The small EVs obtained from umbilical cord blood monocytes have been demonstrated to have no deleterious impact on the viability or metabolic activity of peripheral blood mononuclear cells, THP-1 monocytes, THP-1-derived macrophages, normal dermal human fibroblasts, or human umbilical vein endothelial cells, as well as *in vivo*, they have shown no significant hepatorenal toxicity [172].

At present, the EVs separation technology is immature and requires the establishment of uniform production standards and GMP production protocols that can be prepared in large quantities. EVs are unstable, which means that the vesicles may disintegrate and lose their substances. To become a true drug for lung regeneration, MSC-EVs need to be standardized. To minimize the instability of EVs, the usage of excipients or vibratory mesh nebulizers with larger mesh holes should be considered [173,174]. Harrell and his colleagues designed the MSC-derived product “Exosome-derived Multiple Allogeneic Protein Paracrine Signaling (Exo-d-MAPPS)”, and this product could modulate the pulmonary immune and inflammatory response, and importantly, they have reported no adverse effects after its administration in chronic obstructive pulmonary disease (COPD) patients [175]. Another practical problem to address is the need for a sufficient number of cells. Compared with plastic adherent cultures, three-dimensional (3D) bioreactors help to produce a large number of cells [176,177]. Harazidi et al. have confirmed that 3D hucMSC exosomes produced 20 times more exosomes than the 2D hucMSC exosomes, and 3D hucMSC exosomes produced 7 times more efficient small interfering RNA transfer in targeting organs compared to the 2D hucMSC exosomes [178]. However, other studies also exhibited that 3D cultures could reduce MSCs indoleamine 2,3-dioxygenase (IDO) activity and the EVs significantly reduced macrophage phagocytosis *in vitro* [127]. After the administration of 2D and 3D EVs in bleomycin lung fibrosis mice, it could be found that the EVs did not show benefits for lung injury and pulmonary fibrosis. They concluded that 3D-MSC cultures did not enhance the typical MSC-EV properties, including immunomodulation, antifibrotic, and anti-inflammatory properties, compared to the 2D cultures. Hence, the method of expanding the culture still needs to be further explored.

## 7. Conclusions and Prospects

Exosomes and other EVs are the frontiers of cell-free therapy and nanomedicine research. Communication between cells is essential to maintaining the normal functions of the whole organism and repairing the damaged tissue, which sometimes depends on the paracrine action of cells. Nonetheless, this research is still in its infancy, and many unanswered questions need to be resolved before MSC-EVs become a significant candidate for clinical treatment. MSC-EVs are a comprehensive biologic therapy. It can not only inherit the immunomodulatory, repair-promoting and anti-inflammatory effects of the host cell, but it can also play a targeted role in increasing the accuracy and targeting of the treatment site, making itself a promising strategy for cell-free therapy. In conclusion, it is expected that improving the microenvironment using MSC-EVs will be a future cell-free treatment option to induce lung tissue regeneration and healing in IPF.

## Figures and Tables

**Table 1:** The characteristics of EVs.

Characteristic	Exosomes	Microvesicles	Apoptotic Bodies
Size (nm)	20–150	50–1500	50–2000
Origin	Invagination of cell membrane	Cell membrane budding and fission	Released by cells undergoing apoptosis, plasma membrane, endoplasmic reticulum
Morphology	Cup/round shaped	Various shapes	Heterogeneous
Sucrose gradient	1.13–1.19 g/mL	1.04–1.07 g/mL	1.16–1.28 g/mL
Surface markers	Annexins, tetraspanins, heat-shock proteins	CD40, cholesterol, sphingomyelin, and ceramide	Annexin V positivity, TSP, C3b
Contents	Proteins, enzymes, signal transduction factors, biogenesis factors, chaperones, nucleic acids	Proteins, nucleic acids, lipids	Nuclear fractions, DNA, cell organelles
Isolation technique	Sediment at approximately 100,000 g	Sediment at approximately 10 to 14,000 g	Ultracentrifugation

**Table 2:** Studies of MSC-EVs *in vivo* administration in lung fibrosis.

Number	Reference	Origin	Target Tissue/Model	Year	Administration	Dosage
1	[128]	Human BMSC microvesicles	Lung fibrosis/silica/mouse	2014	Tail vein injection	10 μg
2	[117]	hAEC exosomes	Lung fibrosis/bleomycin/mouse	2018	Intranasal administration	10 μg
3	[129]	AD-MSCs exosomes	Lung fibrosis/silica/mouse	2018	Intratracheal injection	EVs obtained from 100,000 AD-MSCs for 24 h
4	[108]	Human BMSC-EVs/exosomes	Lung fibrosis/bleomycin/mouse	2019	Tail vein injection	200 μL, 8.6–10^8^ particles
5	[126]	MenSC exosomes	Lung fibrosis/bleomycin/mouse	2019	Tail vein injection	0.5 mg/kg/day
6	[124]	Human BMSC exosomes	Lung fibrosis/bleomycin sulfate or silica/mouse	2020	Nebulization	10 × 10^9^ particles/kg
7	[20]	Human BMSC exosomes	Lung fibrosis/LPS/mouse	2020	Tail vein injection	70 μg
8	[130]	hucMSC exosomes	Lung fibrosis/bleomycin/mouse	2020	Tail vein injection	100 μg/250 μL
9	[131]	hucMSC exosomes	Lung fibrosis/silica/mouse	2020	Tail vein injection	-
10	[125]	Human BMSC-EVs	Lung fibrosis/bleomycin/mouse	2020	Tail vein injection	100 μg
11	[132]	Mouse BMSC-EVs	Lung fibrosis/systemic sclerosis/mouse	2021	Intravenous injection	250 ng or 1500 ng
12	[133]	Human BMSC-EVs	Lung fibrosis/bleomycin/mouse	2021	Tail vein injection	100 μg
13	[134]	Rat-BMSC exosomes	Lung fibrosis/silica/rat	2021	Tail vein injection	200 μg/mL/rat
14	[135]	huMSC EVs	Lung fibrosis/bleomycin/mouse	2021	Tail vein injection	20 μg
15	[136]	Human placenta-derived MSC-EVs	Lung fibrosis/radiation/mouse	2021	Tail vein injection	100 μg
16	[127]	Human BMSC-EVs	Lung fibrosis/bleomycin/mouse	2022	Intranasal administration	10 μg
17	[137]	mMSC exosomes	Lung fibrosis/radiation/mouse	2022	Tail vein injection	200 μg
18	[138]	hucMSC exosomes	Lung fibrosis/silica/mouse	2022	-	-
19	[139]	IMRCs EVs	Lung fibrosis/bleomycin /mouse	2022	Intratracheal or tail vein injection	200 μg or 1000 μg

## Data Availability

Data sharing is not applicable to this article as no new data were created or analyzed in this study.
